# The 5-Choice Continuous Performance Test: Evidence for a Translational Test of Vigilance for Mice

**DOI:** 10.1371/journal.pone.0004227

**Published:** 2009-01-19

**Authors:** Jared W. Young, Gregory A. Light, Hugh M. Marston, Richard Sharp, Mark A. Geyer

**Affiliations:** 1 Department of Psychiatry, University of California San Diego, La Jolla, California, United States of America; 2 Schering-Plough Corporation, Newhouse, Lanarkshire, United Kingdom; James Cook University, Australia

## Abstract

**Background:**

Attentional dysfunction is related to functional disability in patients with neuropsychiatric disorders such as schizophrenia, bipolar disorder, and Alzheimer's disease. Indeed, sustained attention/vigilance is among the leading targets for new medications designed to improve cognition in schizophrenia. Although vigilance is assessed frequently using the continuous performance test (CPT) in humans, few tests specifically assess vigilance in rodents.

**Methods:**

We describe the 5-choice CPT (5C-CPT), an elaboration of the 5-choice serial reaction (5CSR) task that includes non-signal trials, thus mimicking task parameters of human CPTs that use signal and non-signal events to assess vigilance. The performances of C57BL/6J and DBA/2J mice were assessed in the 5C-CPT to determine whether this task could differentiate between strains. C57BL/6J mice were also trained in the 5CSR task and a simple reaction-time (RT) task involving only one choice (1CRT task). We hypothesized that: 1) C57BL/6J performance would be superior to DBA/2J mice in the 5C-CPT as measured by the sensitivity index measure from signal detection theory; 2) a vigilance decrement would be observed in both strains; and 3) RTs would increase across tasks with increased attentional load (1CRT task<5CSR task<5C-CPT).

**Conclusions:**

C57BL/6J mice exhibited superior SI levels compared to DBA/2J mice, but with no difference in accuracy. A vigilance decrement was observed in both strains, which was more pronounced in DBA/2J mice and unaffected by response bias. Finally, we observed increased RTs with increased attentional load, such that 1CRT task<5CSR task<5C-CPT, consistent with human performance in simple RT, choice RT, and CPT tasks. Thus we have demonstrated construct validity for the 5C-CPT as a measure of vigilance that is analogous to human CPT studies.

## Introduction

The link between cognitive performance and global functioning/quality of life has been established in numerous disorders including schizophrenia, bipolar disorder, attention deficit hyperactivity disorder, and Alzheimer's disease [1], [2]. Thus there is a need for cognitive therapeutics in the treatment of these disorders, requiring the contribution of industry, academia, and the government to address this ‘great unmet therapeutic need’ [3]. One common cognitive domain that is impaired in each of these disorders is attention/vigilance.

Mackworth [4], pioneered the formal assessment of vigilance in humans, where subjects were required to discriminate between signal and non-signal (noise) stimuli in their environment. This discrimination is often operationally defined as requiring a response to Signals and inhibiting responses to Noise. If this is now considered trial by trial in which there is either a signal or only noise present several possible outcomes are possible. On trials were a Signal is present the subject may correctly respond a “Hit” or fail to respond a “Miss”. On trials were only Noise is present correctly withholding a response is referred to as a “Correct Rejection” (CR) while an erroneous response is a “False Alarm” (FA) (see table 1). In many cases these parameters are expressed as proportions to aid calculation so for example perfect performance would be characterized as a p(Hit) = 1.0, p(Miss) = 0, p(CR) = 1.0 and p(FA) = 0. Over the years authors in the signal detection theory (SDT) area have proposed a series of derived indices based upon these parameters to represent the traits of “Sensitivity” and “Bias”. Sensitivity attempts to quantify the ability of a subject to discriminate a Signal from Noise irrespective of other parameters that may be influencing overall performance [5], [6]. On the flip side “Bias” attempts to quantify the importance and direction that other factors are influencing overall performance such as the various components of response strategies or motivation. Again a series of derived Bias indices are available including perceptual and responsivity bias. To give a flavor of how these may be viewed for a given sensitivity a subject might be either conservative, or liberal, in their response strategy (11, 12). For instance a high p(CR) and low p(FA) matched to a moderate p(Hit) and p(Miss) would indicate a “Conservative” approach of only responding to a signal if you very sure, were as a “Liberal” approach would have a relatively higher p(Hit) and p(FA) which would reflect a strategy of responding to virtually anything that might be a signal.

**Table 1:** Measures used in signal detection theory analyses of performance in CPTs.

	Go Trial	No/Go trial
**Response**	*Hit*	False Alarm
**No Response**	Miss	*Correct Rejection*

*Italicized* responses are rewarded, while non-italicized are punished.

The use of signal detection indices is most common in a range of attention/vigilance tasks that are discussed under the umbrella phrase ‘continuous performance test’ (CPT) [7], [8]. These tasks include the original X-CPT [9], the AX-CPT [10], Connor's CPT [11], and the CPT IP [12]. In each case the aim is to respond on signal trials and inhibit response on noise trials in an experimenter-paced task. Schizophrenics exhibit consistently poorer performance in these tasks compared to controls. As a consequence the Measurement And Treatment Research for Improving Cognition in Schizophrenia (MATRICS) group chose a CPT (the CPT-IP) to be included in the battery for assessing attention/vigilance in schizophrenia patients [13]. Moreover, it has been suggested that poor vigilance, of this type, may represent a core cognitive deficit experienced by schizophrenics, with their inability to attend to their environment possibly being the substrate underlying deficits in higher order integrative cognitive domains [14]–[17]. Thus developing therapeutics for treating this cognitive deficiency is of importance.

Animal modeling of disease processes is a crucial stage in the discovery of treatments to improve the lives of psychiatric patients [18], including those with schizophrenia [19], [20]. Cross-species translatability is vital [21], because animal models provide a degree of experimental control and manipulation opportunities that are not available in human tests [18]. Although several tests of attention in rodents exist, their cross-species translatability could be improved [18], [22], [23]. Such tasks include the 5-choice serial reaction (5CSR) task, first developed by Robbins and colleagues [24], and a sustained attention task validated by McGaughy and Sarter [25]. The 5CSR task has been studied extensively in both rats [26] and mice [27], and requires the rodent to nosepoke wherever a cue light appears in one of 5 spatial locations. It has been suggested that the 5CSR task is analogous to the CPT [28], [29], where incorrect responses (response where no cue is present) in the 5CSR task are analogous to false alarms in the CPT [28]. No explanation is provided for how correct rejections are measured however, despite this being the contrary measurement to false alarms. Thus these interpretations are inaccurate as no non-signal trials are presented in the 5CSR task [26], thus false alarm and correct rejection measurements cannot be generated. Because non-signal stimuli are not presented in the 5CSR task, SDT cannot be used to evaluate performance in a manner consistent with human CPTs. This limitation in turn makes it difficult to compare preclinical performance with equivalent data derived in a human CPT. In this regard, Robbins [30] noted that ‘the test requirements [for the 5CSR task] fall short of that which is normally regarded as vigilance’ (pp. 191). Furthermore, the human version of the 5CSR task for the Cambridge Neuropsychological Test Automated Battery, also developed by Robbins and colleagues, is described specifically as a test of serial choice reaction-time [31], suggesting that further development is required for the rodent 5CSR task to be used as a test of attention/vigilance that translates directly to the human CPT. Another issue derives from the extensive training at a constant cue light onset time (inter-trial interval; ITI), resulting in the subject responding semi-automatically after the completion of the ITI independently of when the cue light is presented. Spratt and colleagues [32] confirmed this suspicion using a rat version of the protocol with non-signal trials interpolated. This behavior would suggest that rats, at least in part, use a temporally mediated strategy to identify when to respond, and do not differentiate responding from non-responding even when no stimuli are present in the traditional 5-CSR task.

The task developed by Sarter and colleagues differs from the 5-CSR task in that rats must attend to a single location to ascertain whether a cue stimulus appears before the response levers are presented. The rat then needs to make a choice to press one lever if it perceived that a stimulus was present, and the other lever if it deems that no stimulus was presented. Although this task measures Hits, Misses, Correct Rejections and False Alarms, it differs from the CPT in that the rat does need to inhibit its response to a non-signal stimulus. In fact, it can be argued that a non-signal stimulus is never actually presented because the rat has to make an active response in every case. The Sarter approach also presents the added difficulty when required to train mice in the task, as mice are more readily trained to nosepoke vs. lever press [33]. In fact to date, there has been only one publication on mice performing this task, which was not validated in terms of vigilance [34]. With the myriad of genetic mouse models of diseases (including schizophrenia), now being developed, the authors felt that adapting the Robbins/Carli approach offers a number of advantages.

**Figure 1:**
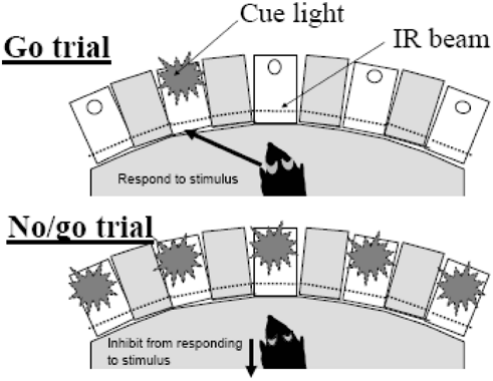
Schematic of the 5C-CPT stimuli. Example of the two trial types in the 5C-CPT. Go trials (relevant stimuli) appear 83% of the time, and the mouse must respond to the stimulus by nose-poking beyond the infra-red (IR) beam in the location of the cue stimulus. Cue stimuli can appear in any one of the five locations. No/go trials (irrelevant stimuli) occur 17% of the time, all five cue lights come on, and the mouse must inhibit from responding in any of the five locations.

Here, we report on the 5-choice CPT (5C-CPT), an elaboration of the 5CSR task that models the task parameters of human CPTs. As in the 5CSR task, mice were trained to respond to signal stimuli (individual lights that could appear in any one of five locations). Consistent with human CPTs however, mice were also required to inhibit responding to non-signal stimuli (lights appearing in all five locations; figure 1). A variable ITI (3–7 s) was also used to limit the potential use of a temporally mediated strategy, and thus extend the period of time the mice must attend to the visual field prior to a stimulus presentation.

We trained standard C57BL/6J and DBA/2J inbred mouse strains in the 5C-CPT to ascertain whether this novel task could differentiate between two strains. 5CSR task performance differences have been observed between these two strains [35], [36], providing a comparison point for results in the 5C-CPT. C57BL/6J and DBA/2J strain differences have been observed in numerous other cognitive tasks as well [37]–[39]. Thus by using SDT from the 5C-CPT we may determine whether these cognitive deficits are attentional in nature, or whether the poor performance of DBA/2J mice are confounded by strategy bias' [40]. The latter, DBA/2J strain, has been shown to exhibit lower α7 nicotinic receptor expression as well as poorer sensory gating ability. These differences suggest that it may, in part, mimic some of the attributes that dissociate schizophrenics from normal individuals [52], [53]. Due to reinforcement deliveries following accurate performance, the 5C-CPT resembles self-paced human CPTs, which remain sufficient to differentiate between schizophrenia patients and normal controls (Neuchterlein personal communication). Given the consummatory phases that are inherent in the 5C-CPT and not present in experimenter-paced human tasks however, the 5C-CPT also requires validation as a test of vigilance. Therefore, the performance of the two strains in an extended session task, hypothesizing that both strains would exhibit a vigilance decrement in the 5C-CPT consistent with human CPT performance [41]. Further, to probe the effects of different attention al load C57BL/6J mice were trained in 5-choice and 1-choice (1CRT) versions of the task. It was further hypothesized that, consistent with humans [41], there would be an inverse relationship between response time and increasing attentional load such that response time would be fastest in the 1CRT task, intermediate in the 5CSRT task, and slowest in the 5C-CPT.

## Results

### Baseline 5C-CPT strain comparison

**Figure 2:**
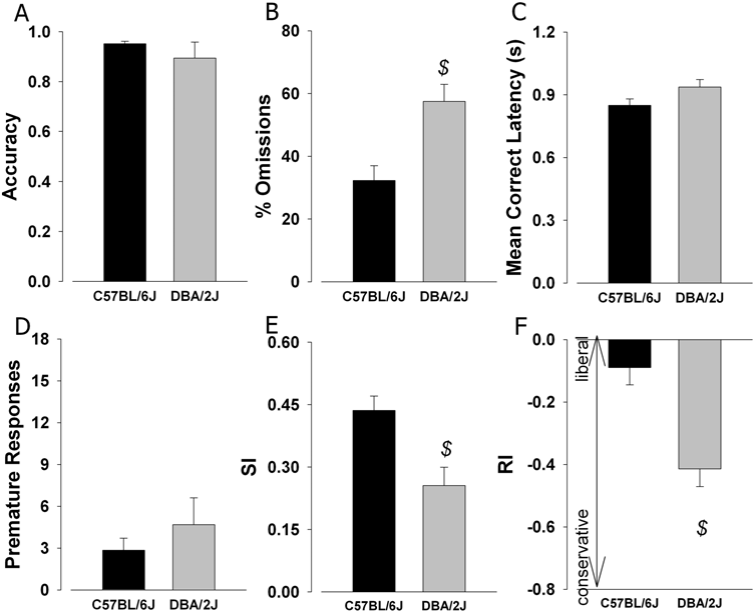
Strain performance at baseline in the 5C-CPT. Performances of C57BL/6J and DBA/2J mice were compared at baseline in the 5-choice continuous performance test (5C-CPT). The 5C-CPT was adapted from the 5CSR task and includes no-go trials, requiring greater stimulus and inhibitory control. The two strains of mice demonstrated equal ability in discriminating between target locations as measured by accuracy (A). A trend towards greater %Omissions in DBA/2J mice was observed however (B). No significant strain effects on mean correct latency (C) or premature responses (D) were observed. The inclusion of no-go trials in the 5C-CPT allowed the use of signal detection theory, with which the sensitivity index (SI) could be calculated as an index of vigilance performance, consistent with human CPTs. Differences in SI were observed between the two strains (E), although this was not significant. SDT was also used to calculate responses index (RI) bias (F). There were trends toward poorer SI levels and more conservative responding in DBA/2J mice. Data presented as mean+s.e.m., and *$* denotes *p*<0.1 when compared to C57BL/6J mice.

Once trained in the 5C-CPT, the performance of C57BL/6J and DBA/2J mice was compared in the standard 120-trial test session. Standard 5CSR task performance measures as well as the novel measures from the 5C-CPT were compared. No significant effect of strain on the traditional 5CSR task attentional measure, accuracy [26], was observed (F(1,5) = 1.0, NS; Fig 2A). There was a trend towards increased %Omissions in DBA/2J compared to C57BL/6J mice (F(1,5) = 5.0, p = 0.076; Fig 2B). No effect of strain was observed on MCL (F(1,5) = 2.1, NS; Fig. 2C) or premature responses (F(1,5) = 0.5, p = 0.5; Fig. 2D). In the 5C-CPT measures of sensitivity, no significant effect of strain was observed on SI (F(1,5) = 4.8, *p*<0.1; Fig. 2E). There were trends towards increased bias RI (F(1,5) = 5.6, p = 0.064; Fig. 2F) in DBA/2J compared to C57BL/6J mice. Thus, when mice are fully trained in the 5C-CPT, performance did not differ significantly between strains, although several trends were evident. Performance was therefore challenged by increasing the trial number to 250 trials, thereby increasing the attentional load placed on the mice, as well as allowing assessment of performance decrements across the session.

### Extended session 5C-CPT strain comparison

**Figure 3:**
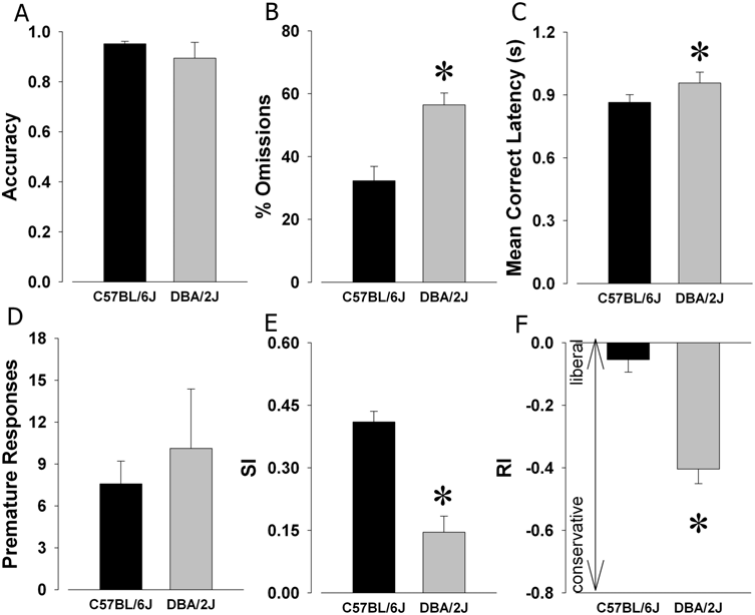
Strain performances in the 5C-CPT extended session. 5C-CPT performances of C57BL/6J and DBA/2J mice were compared in an extended session challenge (250 trials), to increase attentional load. Consistent with baseline performance, the two strains of mice demonstrated equal ability in discriminating between target locations as measured by accuracy (A). DBA/2J mice exhibited significantly higher levels of %Omissions (B), and a slower mean correct latency (C), compared to C57BL/6J mice however. The challenge did not result in differences in premature responses (D), but significant differences in vigilance performance SI (E) was observed between the two strains, with poorer performance in DBA/2J mice. The DBA/2J mice also exhibited a significantly more conservative response bias compared to C57BL/6J mice, based on responsivity index (RI; H). Data presented as mean+s.e.m., and * denotes p<0.05 when compared to C57BL/6J mice.

The mice were tested on an extended session challenge. The protocol of the task remained the same to avoid possible learning confounds, but the number of trials was increased to 250 trials. Even with increased attentional load, no significant effect of strain was observed for accuracy (F(1,15) = 0.7, NS; Fig 3A). Significant effects of strain were observed for %Omissions (F(1,15) = 12.6, p<0.005; Fig 3B) and MCL (F(1,15) = 6.3, p<0.05; Fig 3C), as C57BL/6J mice exhibited lower levels of %Omissions, and faster MCL than DBA/2J mice. No significant effect of strain was observed for premature responses (F(1,15) = 0.3, NS; Fig. 3D). Several of the measures unique to the 5C-CPT yielded significant main effects of strain however. Significant strain effects for SI (F(1, 5) = 19.0, p<0.01; Fig 3E) were observed, with C57BL/6J mice exhibiting greater performance than DBA/2J mice. Finally, a significant effect of strain was observed for RI (F(1,5) = 7.6, p<0.05; Fig. 3F), with lower values for DBA/2J compared to C57BL/6J mice for both measures, indicative of a more conservative response bias in DBA/2J mice. No significant effect of strain on B″ was observed (F(1, 5) = 2.5, NS), indicating a lack of difference in perceptual bias between the two strains.

### Extended Session performance analysis–within task assessment

#### Performance across Trial Bins in the 5C-CPT

**Figure 4:**
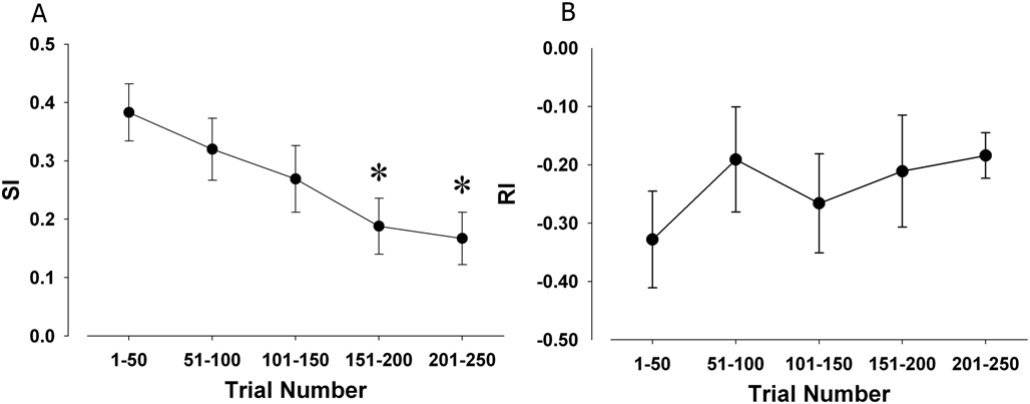
Mouse vigilance decrement in the 5C-CPT over time. The performances of C57BL/6J and DBA/2J mice over time in the 5C-CPT were compared. Performance was binned into 50 trial blocks to ensure consistency in trial number across blocks so that proportional data could be compared. No strain by trial block interaction was observed for any measure. A significant main effect of trial block was observed for SI (A) however, indicative of poorer vigilance with cognitive fatigue. No effect of trial block was observed for responsivity index RI (B), indicating that the deterioration of cognitive performance over time was not confounded by physical factors, consistent with human CPT performance. Data presented as mean+s.e.m., and * denotes *p*<0.05 when compared to trial block 1.

Performance of mice in the 5C-CPT was binned into five 50-trial bins (1 = 1–50, 2 = 51–100, 3 = 101–150, 4 = 151–200, 5 = 201–250) and compared for each measure. In the traditional measure of attentional performance in the 5CSR task, accuracy, a significant effect of trial bin was observed F(4,20) = 4.2, p<0.05), with no trial bin by strain interaction (F(4,40) = 1.1, NS). Post hoc analyses revealed that trial bin 5 differed significantly from bins 2 and 3 (p<0.05), although no significant differences between any other trial bins were observed (p>0.05). There were trends towards both an effect of trial bin (F(4,20) = 3.0, p = 0.086) and an interaction between gene and trial bin (F(4,20) = 2.8, p = 0.099) on premature responding. In the sensitivity measure SI, a significant main effect of trial bin was observed (F(4,20) = 4.4, p<0.01; Fig. 4A), with no trial bin by strain interaction (F(4,20) = 1.6, NS). Post hoc analyses revealed that performance at trial bin 1 and 2 was significantly greater than performance in trial bins 4 and 5, and that performance in bin 3 was greater than that in bin 4 (p<0.05). No significant main effects of trial bin on bias measures RI (F(4,20) = 1.4, NS) or B″ (F(4,20) = 2.0, NS; Fig. 4B) were observed, indicative of no change in response or perceptual bias over time in the task.

#### Performance across ITI bins in the 5C-CPT

**Figure 5:**
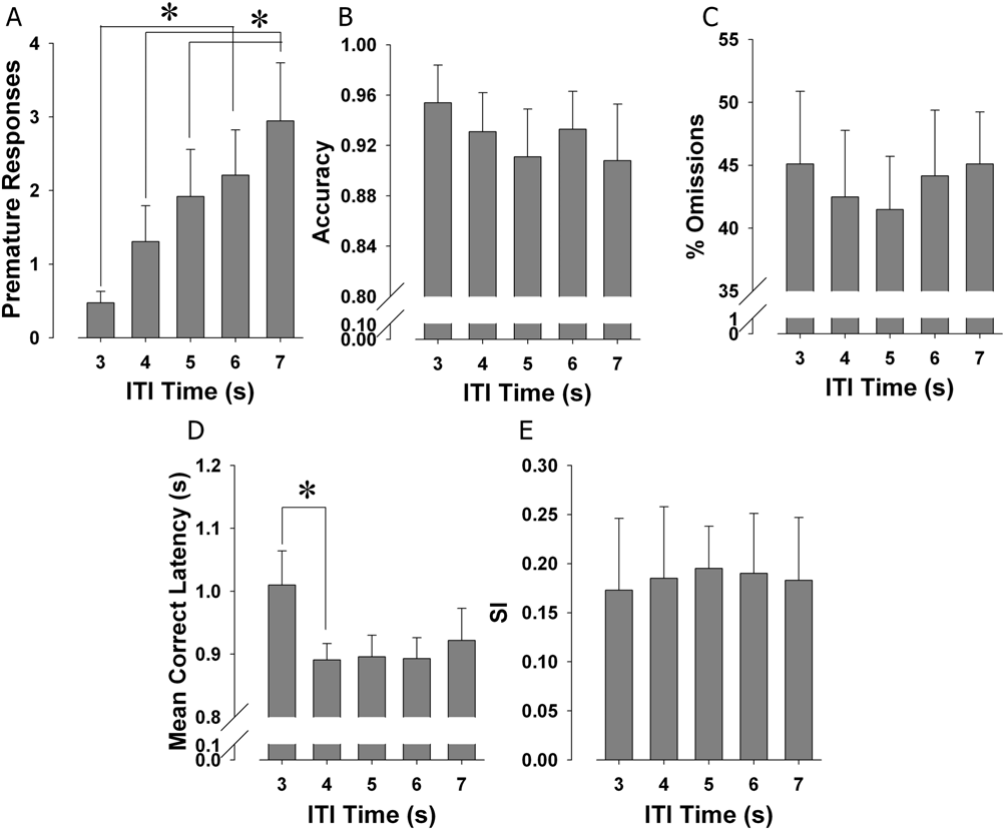
5C-CPT extended session performance of mice binned by ITI time. The 5C-CPT included a variable inter-trial interval (ITI; variable period after which the cue stimulus can appear), ranging from 3–7 s. Performance of the two strains was binned according to ITI time and compared, to assess the effects of ITI bin on performance. No interaction between strain and ITI time was observed for any measure. Increased premature responses were observed with increasing ITI time (A), demonstrating the temporal impulsivity resulting from consistent training in this variable ITI. No significant effects of ITI time on accuracy (B) were observed, nor on %Omissions (C). A significant main effect of ITI time on RT was observed, with performance being fastest at the middle ITI times. No significant main effects of ITI time on SI (F) were observed, although, consistent with %Omissions and RT, the best performance appeared at the center ITI times. Data presented as mean+s.e.m., and * denotes p<0.05 when compared to ITI time indicated.

During extended session performance in the 5C-CPT, data were also binned according to ITI time (3, 4, 5, 6, or 7 s) and compared. Significant main effects of ITI bin was observed for premature responses (F(4,20) = 6.8, p<0.01; Fig. 5A), with no ITI bin by strain interaction (F(4,20) = 0.4, NS). *Post hoc* analyses revealed significant increases in premature responding with increasing ITI times - premature responses at ITI times 5, 6, and 7 were each significantly more when compared to ITI time 3 (p<0.05). ITI time 7 also differed significantly from ITI time 4 (p<0.05). No main effects of accuracy (F(4,20) = 2.7, NS; Fig. 5B) or ITI bin by strain interactions (F(4,40) = 1.4, NS) were observed. No main effect of ITI bin (F(4,20) = 0.5, NS) or ITI bin by strain interaction (F(4,40) = 0.4, NS) was observed for %Omissions (Fig. 5C). A significant main effect of ITI bin on MCL was observed (F(4,40) = 3.1, p<0.05; Fig. 5D), but no ITI bin by strain interactions (F(4,40) = 2.1, NS) were observed. Post hoc analyses revealed that MCL at ITI time 4 was faster than at ITI time 3 (p<0.05). No main effect of ITI bin on SI (F(4,20) = 0.1, NS; Fig. 5E) was observed, nor were there ITI bin by strain interactions for SI (F(4,20) = 1.6,NS). No main effect of ITI bin on response bias differences RI (F(4,20) = 1.2, NS) was observed.

#### Performance measures generated from non-responses in Go versus No-go trial-types

**Figure 6:**
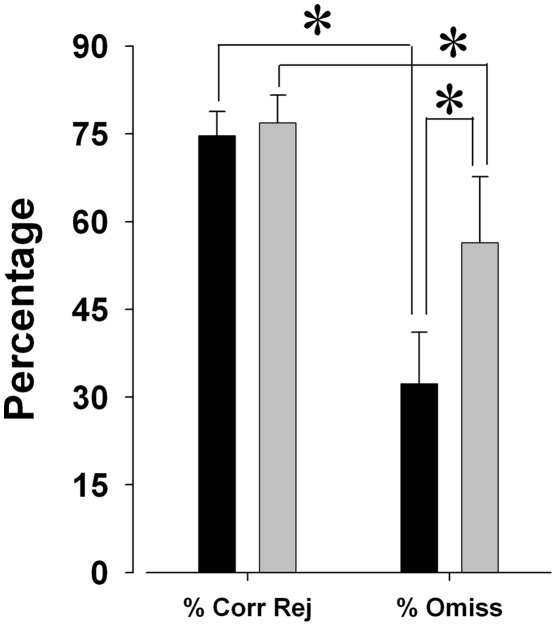
Comparison of percentage non-responding in no-go vs. go trials in the 5C-CPT. Given the similarity in response type for a correct rejection in a no-go trial to a miss in a go trial (both are represented by a lack of response), it is important to establish that the performance of the mice is dependent upon trial type. C57BL/6J and DBA/2J mice exhibited comparable levels of %Correct Rejection (%Corr Rej) during no-go trials in the extended session 5C-CPT. During go trials however, C57BL/6J mice exhibit significantly lower levels of %Omissions compared to DBA/2J mice. Importantly, both groups exhibited significantly different levels of non-response to each trial type, suggesting that they performed differently during go trials compared to no-go trials, and hence they their response was dependent upon trial type. Data presented as mean+s.e.m., and * denotes *p*<0.05.

Given that an omission error in a go trial results from the same behavior as a correct rejection in a no-go trial (i.e. lack of response to a cue stimulus), %Correct Rejections from no-go trials were compared to %Omissions from go trials (from the extended session) to assess whether mice treated the two trial types the same. Significant differences between %Correct Rejection and %Omissions were observed for C57BL/6J (F(1,17) = 66.2, *p*<0.0001; Fig 6) and DBA/2J mice (F(1,12) = 7.7, *p*<0.05; Fig 6). The effect sizes for C57BL/6J and DBA/2J mice were 0.796 and 0.391 respectively, suggesting a greater differentiation between scores for C57BL/6J compared to DBA/2J mice.

### 5C-CPT, 5CSR task, and 1CRT task: Baseline task comparison

**Figure 7:**
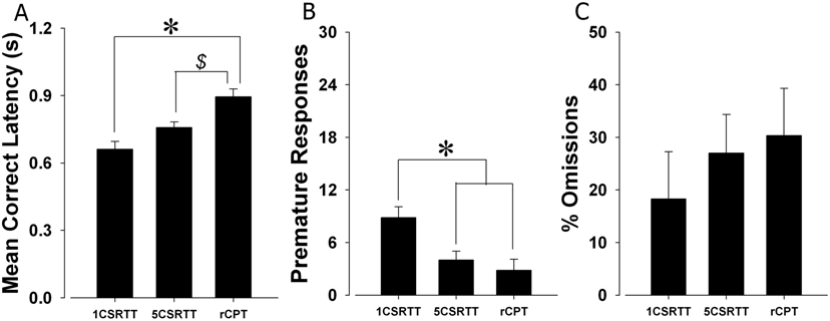
Performance of C57BL/6J mice across simple and choice RT tasks as well as in the 5C-CPT at baseline. Three separate groups of C57BL/6J mice were trained in a simple (1CRT task) and choice (5CSR task) RT tasks as well as the 5C-CPT. Significant differences in performance were observed across tasks, where consistent with human performance, RT was fastest in a simple RT task, and slowest in a vigilance task (A). Performance also differed as measured by premature responses, with the greatest levels observed in the simple RT task requiring the lowest level of inhibitory control (B). Increased %Omissions were observed with increased attentional load, although these effects were not significant (C). Data presented as mean+s.e.m., * denotes *p*<0.05 and *$* denotes *p*<0.1 when compared to tasks indicated.

Baseline performances (maximum of 120 trials) of C57BL/6J mice in a simple RT task, choice RT task, and the 5C-CPT were compared. A significant main effect of task type on RT was observed (F(2,11) = 7.3, p<0.01; Fig 7A). Post hoc analyses revealed a significant MCL difference between 5C-CPT and 1CRT task performance, but only a trend towards significance between the 5C-CPT and 5CSR task (p = 0.066), and the 1CRT task and 5CSR task (p = 0.054). A significant main effect of task was observed for premature responding (F(2,11) = 6.8, p<0.05; Fig 7B), with Tukey post hoc analyses revealing that premature response levels in the 1CRT task were higher compared to both 5CSR task and 5C-CPT (p<0.05), while levels in the 5CSR task did not differ from the 5C-CPT (p>0.05). No significant main effect of task was observed in %Omissions (F(2,11) = 0.04, NS; Fig. 7C). Consistent with the studies reported above, performance was then challenged by extending the session length to 250 trials, thus increasing the attentional load.

### 5C-CPT, 5CSR task, and 1CRT task: Extended session task comparison

**Figure 8:**
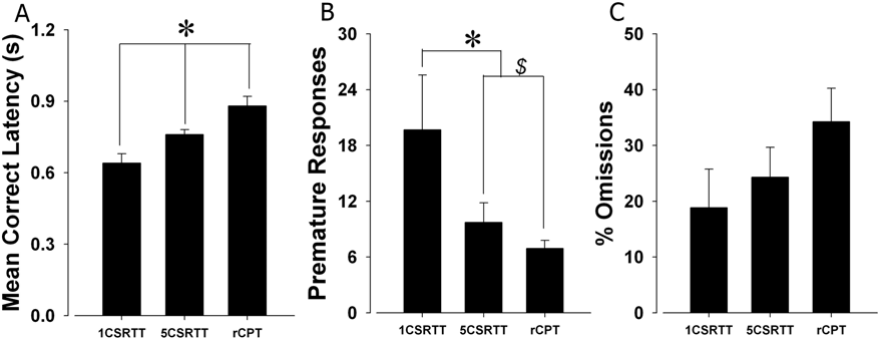
Performance of C57BL/6J mice across simple and choice RT tasks as well as in the 5C-CPT in the extended session. The performance of three separate groups of C57BL/6J mice was compared with an extended session challenge, in simple (1CRT task) and choice (5CSR task) RT tasks, as well as the 5C-CPT. Significant RT differences were observed across tasks, where consistent with human performance, RT was fastest in a simple RT task, intermediate in the choice RT task (5CSR task), and slowest in a vigilance task (5C-CPT; A). A significant difference in premature responses, with the greatest levels being observed in the simple RT task that required the lowest level of inhibitory control (B). Increased %Omissions were observed with increased attentional load, although these effects were not significant (C). Data presented as mean+s.e.m., * denotes p<0.05 and $ denotes p<0.1 between tasks indicated.

We investigated whether performance of C57BL/6J mice would differ relative to the attentional loads associated with the different cognitive tasks (1CSR task<5CSR task<5C-CPT), consistent with humans in simple and choice RT tasks or in the human CPTs [41]. Significant main effects of attentional load were observed for several measures. As before, a significant main effect of task type on MCL was observed (F(2,11) = 16.5, p<0.0001; Fig 8A), with post hoc analyses revealing that MCL was dependent upon task difficulty. MCL was again faster in the 1CRT task compared to the 5CSR task (p<0.05) and MCL in both tasks were faster than the 5C-CPT (p<0.05) in this extended task challenge. A significant main effect of task was also observed for premature responding (F(2,11) = 4.2, p<0.05; Fig 8B), with Tukey post hoc analyses revealing that, consistent with baseline, premature response levels in the 1CRT task were higher compared to both 5CSR task and 5C-CPT (p<0.05), while now there was a trend of increased levels in the 5CSR task compared to the 5C-CPT (p<0.1). Again, no significant main effect of task was observed in %Omissions (F(2,11) = 1.5, NS; Fig. 8C), although a greater separation from baseline performance between the three tasks was observed with %Omissions increased with increasing attentional load.

## Discussion

The present studies describe the 5-choice continuous performance test (5C-CPT), an evolution of the 5CSR task designed to assess vigilance in mice with task parameters consistent to those used in human testing. The data presented confirm that mice can; be trained to perform the 5C-CPT, discriminate between signal and non-signal stimuli, and exhibit a vigilance decrement over time, Further, the evolution of the experimental designed allowed the effective deployment of signal detection parameters within the analysis of the data. These parameters both facilitated the identification of strain dependent response strategies as well offered an improved ability to draw analogies with similarly derived clinical data. As predicted, DBA/2J mice, though almost as accurate, exhibited generally poorer performance compared to C57BL/6J mice. This was exemplified by the DBA/2J mice having poorer sensitivity in detecting the signal stimuli (reduced SI), a more conservative response strategy (increased RI) and a higher omission rate. Thus, the 5C-CPT does appear to assess attention with task parameters that are consistent with the cognitive construct of vigilance

Initial construct validation of the 5C-CPT to the human CPT in assessing vigilance is apparent from the present studies. Consistent with human vigilance studies [41], a vigilance decrement was observed in both strains of mice (C57BL/6J or DBA/2J). This decrement was observed most clearly in the extended session version of the task in the sensitivity index (SI; Fig. 4A). Indeed SI was the only index of performance to show a classical progressive fall off with time that was significant from the middle block of trials. By contrast, accuracy only showed a clear effect in the last block in trials and that only in comparison with the second block after a slight increase in accuracy from the first block [26]. Furthermore, because the 5C-CPT enabled the generation of responsivity bias indices, the vigilance decrement observed could be differentiated from response bias' differences over time (Fig. 4B), again consistent with human CPTs. Because response bias did not change over time (Fig. 4B), the vigilance decrement observed was likely to be mediated by cognitive as opposed to motivational factors [40]–[42]. Also consistent with human performance tasks, response was fastest in a simple RT task (1CRT task), intermediate in a choice reaction time task (5CSR task), and slowest in a vigilance task (5C-CPT; [43], [44]; Fig. 8A). Thus, the 5C-CPT appears to fulfill criteria as a test of vigilance based on these parametric manipulations of attentional load [41]. It must though be acknowledged that in this task we can only measure response time, not reaction time as the time to move is included. This may confound the difference between the 1CSR and the 5CSR and 5C-CPT tasks where the subject may have significantly further to move to make a response in one of the five, as opposed to one, possible response locations. This however cannot account for the difference between the latter two more complex tasks where the distances are no different.

Given that the present studies demonstrate orderly 5C-CPT performance in mice, this task appears to be suitable to assess the effects of genetic manipulations and/or genetic models of psychiatric disorders on attentional performance. Further, the present data demonstrate that performance between strains can be differentiated effectively using the 5C-CPT. In contrast to observed differences using SDT measures, simple accuracy [26] did not differentiate performance of C57BL/6J and DBA/2J mice in the 5C-CPT. This lack of difference in accuracy between these strains contrasts with previous reports using the 5CSR task [35], [36], which could reflect the greater inhibitory control required in the 5C-CPT. Thus, DBA/2J mice may be exhibiting greater stimulus response control in the 5C-CPT, perhaps as a result of the inclusion of non-signal events (noise trials) that require the inhibition of responding. By contrast, DBA/2J mice exhibited significantly higher rate of omissions than C57BL/6J mice in the 5C-CPT and although omission levels may reflect motivational influences [26], numerous 5CSR task studies report that with increased attentional load omission levels may be sensitive measures of attentional performance [45]–[50]. This conclusion is support by the concomitant reduction in SI seen in the DBA/2J mice when tested in the extended version of the 5C-CPT.

The use of non signal trials and SDT in the 5C-CPT also enables the assessment of response bias. Bias measures provide information on whether a manipulation may have altered a group's perceptual or response bias/strategy, as opposed to affecting attentional processes. These measures can be especially important in a task that has a heavy locomotor component and provides rewards and ‘punishments’, because genetic and pharmacological manipulations may affect these behaviors differentially from attention. The poorer vigilance performance of DBA/2J mice may have been due partially to their more conservative response strategy as measured by RI. Thus DBA/2J mice were more likely to withhold responding than C57BL/6J mice. These results highlight the importance of SDT when assessing attentional functioning, since it enables one to identify whether putative attentional deficits [35], [36] may be due to the use of different response strategies rather than alterations in attention. These findings also emphasize the need for nonsignal events in the 5CSR task and that their lack limits the analogy between the 5CSR task and the CPT [28]. Furthermore the strain differences observed in the self-paced 5C-CPT are consistent with comparisons of schizophrenia patients to healthy controls in experimenter-paced CPTs [51]–[53], who also exhibit lower d′ and a more conservative response bias. Also consistent with schizophrenia subjects relative to healthy controls is that DBA/2J mice exhibit lower expression of the α7 nicotinic acetylcholine receptor (nAChR) compared to C57BL/6J mice [54], [55]. Mice with reduced α7 nAChR expression on a C57BL/6J background strain trained in the 5-CSR task also exhibit increased omission levels [49], [50], suggesting that differences in expression of this receptor in DBA/2J mice may mediate their poor performance in the 5C-CPT. Alternatively, the dopaminergic system differs between these strains [56], which has been linked to executive control and the formation of strategies [57]. Given the response strategy differences of these two strains identified in the present studies, a dopaminergic mediation cannot be discounted as contributing to the difference in performance. The more conservative response strategy in DBA/2J mice was unlikely to reflect reduced motivation, because the latency to collect food rewards did not differ between strains, consistent with previous reports. Moreover DBA/2J and C57BL/6J mice exhibited similar motivational levels as assessed by work-rate to gain a single reward in a progressive ratio breakpoint study [58]. Thus, while the underlying mechanisms of strain performance differences in 5C-CPT have yet to be elucidated, the use of SDT not only provides information on the attentional performance of mice, but also on possible differences in response strategy, providing further information on any manipulation-induced changes that may occur.

SDT has been used in the analyses of other rodent models of cognition in the past [59]–[61] in part to offer an enhanced level of interpretation to preexisting protocols [40]. Task parameters in these previous animal cognitive studies differed from those of a CPT however, and hence these studies did not measure vigilance consistent with human testing, as operationally defined by Mackworth [4] and Rosvold, [9]. For example, false alarms in Dudchenko et al, [59] were recorded for responses prior to a signal, as opposed to in response to a non-signal event. Also, in the study by Steckler et al [60], false alarms represented incorrectly remembered lever responses after a delay, so that SDT analysis on these data reflects memory as opposed to vigilance performance [40], [62]. To date, the closest rodent animal analogue of the CPT is the task developed by Bushnell, Sarter, and colleagues [25]. These tasks effectively require a rat to respond on one lever if a signal is detected, but respond on another if the rat perceived no signal to be presented. This task differs from human CPT tasks insofar as it requires a response to a stimulus in one location and a response in a different location when the rat perceives that a stimulus did not appear, while CPT tasks provide the human with stimuli to respond to, and different stimuli that require an inhibition of responding [8]. Nevertheless, Sarter and colleagues have used SDT successfully to provide a sensitivity measure of rat performance based on SI, which they termed Vigilance Index (VI: [25]). It is interesting to note that mice in the 5C-CPT produced levels of SI that are comparable to rat performance as measured by VI in the task of Sarter and colleagues [25].

The protocol for the 5C-CPT also benefits from assaying inhibitory control in rodents. This control is measured in response to stimuli that are presented, but are irrelevant, consistent with clinical testing of impulsive responding. This false alarm measure of response to an irrelevant stimulus contrasts with a false alarm response generated in the task of Sarter and colleagues, which occurs when rats respond at a lever when no stimulus was presented. Furthermore, the traditional measure of impulsivity in the 5CSR task is when responses are made prior to the stimulus appearing (i.e. premature responses [26]). This measure captures only one facet of impulsivity [63]–[65], while it has been demonstrated that multiple aspects can be differentiated in animal studies [66]. Thus false alarms in the 5C-CPT may provide a measure of impulsivity [63] that is more consistent with human CPTs [8] than are premature responses in the 5CSR task, and therefore more relevant to clinical testing. Moreover, ongoing studies suggest that false alarm rates and premature responses are affected differentially by some manipulations (Young et al, unpublished observations). The 5C-CPT provides the opportunity to assess both forms of impulsivity within the same task.

Further validation of the 5C-CPT is required however. Numerous studies have investigated the construct validity of the 5CSR task in rats and mice [14], [26], with respect to assessing sustained attention. For example, the prefrontal cortex mediates sustained attention/vigilance in humans [67], [68], consistent with frontal lesions impairing rat performance of the 5CSR task [69]. The parietal cortex is also important for human sustained attention/vigilance however, but is not involved in human choice RT tasks, nor is it required for rat performance of the 5CSR task [69]. The lack of parietal cortical requirement for 5CSR task performance is likely due to every trial requiring the same ‘go’ response [26], for the parietal cortex is involved only when multiple stimuli appear each requiring a different response, as it executes a ‘matching function’ for stimulus to response [70]–[72]. Thus, the parietal cortex is activated during human CPTs testing because relevant and irrelevant stimuli are presented requiring either a response or non response [8]. It has been hypothesized that an inability to ignore irrelevant stimuli leads to vigilance dysfunction in neuropsychiatric patients, making assessment of inhibitory control in response to irrelevant stimuli vital. The 5C-CPT requires the rodent to ignore irrelevant stimuli and as it requires the shifting from one stimulus-response association to another (i.e. stimulus-inhibition consistent with the human CPTs), it is hypothesized that the parietal cortex will mediate performance in this task, in contrast with the 5CSR task [69].

Pharmacological predictive validation of this task could be assessed by investigating the effects of psychostimulants on performance. Methylphenidate improves d′ in children [73] and normal adults [74], while amphetamine has also been shown to improve vigilance in young and old, normal and ADHD children as measured by d′ [75], [76]. In non-smoking normal adults, nicotine significantly improved attentional capabilities in the human CPT as measured by reduced levels of omission, increased hit rate, and reduced RT [77]. Although d′ was not calculated, an improvement in hit rate with no effect on false alarms could suggest an increased d′ in these control subjects [78]. Thus the effects of methylphenidate, amphetamine, and nicotine on d′ could be investigated to further validate the 5C-CPT as a translatable model of sustained attention/vigilance. The evidence of a vigilance decrement in this task, as well as differential performance within ITI bins, also provides a window in which putative cognition-enhancing drugs could be assessed.

One limitation of the 5C-CPT protocol in relation to standard CPT tests is that it is self- and not experimenter-paced. Each trial begins automatically after the mouse collects the food reward at the magazine, or simply nose-pokes in the magazine after an error. To examine the importance of this difference, mice could be trained to perform the task in groups of trials (e.g. 20), with correct responses indicated by a secondary reinforcer that would not require a consummatory response, with primary rewards being delivered at the end of the group of trials. Despite this protocol difference however, the present studies provide construct validity for the 5C-CPT as a test of vigilance that parallels human CPTs, given the demonstration of a vigilance decrement that is unaffected by bias or strain differences in levels of vigilance, as well as reaction time differences that correspond to increases in attentional loads [41]. Furthermore, psychiatric groups exhibit impaired CPT performance even in self-paced tasks. Schizophrenia patients exhibit lower d′ levels compared to healthy controls in a self-paced CPT, developed by Neuchterlein and colleagues (*personal communication*). Thus, while further validation for the 5C-CPT as a test of vigilance is still required, the authors believe that the 5C-CPT provides an opportunity to assess vigilance in mice, in a manner relevant to testing conducted in psychiatric populations.

In summary, the 5C-CPT paradigm described here provides a means with which to assess vigilance in rodents in a comparable form to the CPT used in humans. Observation of a vigilance decrement and RT differences in the three tasks across varied attentional loads support the conclusion that the 5C-CPT assesses vigilance performance. The data presented provide evidence of poorer vigilance performance in DBA/2J mice when compared to C57BL/6J mice, as well as a more conservative response bias. In the context of translational drug discovery, it may therefore be likely that effects of putative cognitive enhancers observed in the 5C-CPT would exhibit substantial cross-species predictive validity for effects in the human CPTs.

## Methods

### Animals

Male C57BL/6J and DBA/2J mice were obtained from Jackson Laboratory (Bar Harbor, Maine). Training began at approximately 3 months of age, with mice weighing between 20–40 g. Mice were housed in groups of maximum 4/cage. Mice were maintained at 85% of free-feeding weight, with water available *ad libitum*, and housed in a vivarium on a reversed day-night cycle (lights on at 8.00 PM, off at 8.00 AM). Mice were brought to the laboratory 60 min before testing between 9.00 AM and 6.00 PM. All procedures were approved by the UCSD Institutional Animal Care and Use Committee. The UCSD animal facility meets all federal and state requirements for animal care and was approved by the American Association for Accreditation of Laboratory Animal Care.

### Apparatus

Training and testing took place in four 5-hole operant chambers (25×25×25 cm, Med Associates Inc., St. Albans, VT). Each chamber consisted of an array of five square holes (2.5×2.5×2.5 cm) arranged horizontally on a curved wall 2.5 cm above the grid floor opposite a food delivery magazine (Lafayette Instruments, Lafayette, IN) at floor level and a house-light near the ceiling. The chamber was located in a sound-attenuating box, ventilated by a fan that also provided a low level of background noise. An infra-red camera installed in each chamber enabled the monitoring of performance during training and testing. Mice were trained to respond with a nose-poke to an illuminated LED recessed into the holes. Responses were detected by infrared beams mounted vertically located 3 mm from the opening of the hole. Liquid reinforcement in the form of strawberry milkshake (Nesquik® plus non-fat milk, 30 µl) was utilized was delivered by peristaltic pump (Lafayette Instruments, Lafayette, IN) to a well located in the magazine opposite the 5-hole wall. Magazine entries were monitored using an infrared beam mounted horizontally, 5 mm from the floor and recessed 6 mm into the magazine. The control of stimuli and recording of responses were managed by a SmartCtrl Package 8-In/16-Out with additional interfacing by MED-PC for Windows (Med Associates Inc., St. Albans, VT) using custom programming (performed by RS).

### Training

Mice were trained in the 5CSR task as described previously [50], with each session lasting 30 min or 120 trials, whichever was completed first. Each trial was initiated by the mouse nose-poking, then removing its nose, from the magazine. Following a variable 3–7 s ITI, a light stimulus appeared in one of the 5 apertures located opposite the magazine. A nose-poke in the lit aperture during the stimulus plus a 2 s limited hold period resulted in a **correct** (Hit) response being registered and a reward being delivered in the magazine. A nose-poke in any other aperture over this period was registered as an **incorrect** response and resulted in a 4 s time-out. Failure to respond in any aperture during the stimulus plus limited hold was registered as an **omission** (omission+incorrect = Miss) and also resulting in a time-out. Response in any aperture during the ITI registered a **premature** response and triggered a time-out. The next trial began when the mouse entered, then exited the magazine. The SD began at 20 s and was reduced to 10, 8, 4, 2, and 1.5 s following the attainment of each criterion (a mean correct latency of less than half of the current stimulus duration for two consecutive days) across sessions. Halfway through training, some C57BL/6J mice (n = 4) were moved to the 1CRT task paradigm which was identical to the 5CSR task except that stimuli are only presented in the center hole. Training in the 5C-CPT was similar to that of the 5CSR task. For the 5C-CPT however, while 100 trials were go (signal) trials, identical to trials described in the 5CSR task where the cue stimulus could appear in any 1 of the 5 apertures, 20 trials were no-go (non-signal) trials, unique to the 5C-CPT where all 5 apertures were illuminated and the mouse had to inhibit responding (see Fig. 1). Training in each task took approximately 5 months. Consistent with human CPTs [8], successful inhibition of a response in a no-go trial resulted in a **correct rejection** (CR) being recorded and reward delivered. A response in a no-go trial however, resulted in a **false alarm (FA)** being registered and a time-out occurring. These no-go trials were pseudo-randomly interspersed within the 100 go trials (maximum of 3 sequential no-go trials). **False alarm latency** was also recorded.

For all three tasks, the **mean correct latency (MCL)**, **mean incorrect latency (MIL)** and **mean premature latency (MPL)** were calculated along with the following parameters.
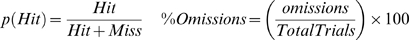


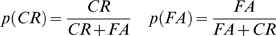



Based upon these basic parameters SDT indicies [5], [6] were then calculated to assess both sensitivity, **SI**, and bias, **RI**, and **B″**.

The premise of signal detection theory is that for a given external stimulus, if repeated, the internal representation of that stimulus will result in a distribution of perceived stimulus strengths. This distribution will have a mean and a standard deviation. If this stimulus is going to be detected as a signal, different from the noise, then the mean of the signal distribution has to be statistically different from the distribution of representations elicited by noise events. Thus the simplest representation of d′ is:
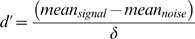
where δ is the standard deviation of the distribution. As such the index is parametric and to be properly used needs to conform to the assumptions of normality, homogeneity of variance and absence of skew. If this can not be confirmed then it is proper to default to a non-parametric approach of which there are several [40], [62], [79]. In this case SI [62] has been chosen as the measure of sensitivity, being non-parametric less assumptions over the nature internal perceptions of the signal need to be made. Further, due to the model used to derive the parameter it is better suited than other available indices to separating performance that clusters at the high end of the scale as is the case here.

The sensitivity index (SI), was calculated using the following formula:
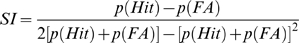
SI provides a non-parametric assessment of sensitivity [62]. Values for SI vary from −1 to +1, with +1 indicating that all signal events were responded to, while all non-signal events were inhibited from responding to, while zero indicates chance levels of distinguishing between signal and non-signal events. SI was also the basis by which Sarter and colleagues [25], developed their vigilance index measure and so would produce comparable results for mice to those seen in rats performing their vigilance paradigm.

To mirror the use of SI the non-parametric response bias measure RI [62] was chosen to provide a measure of the “tendency to respond” [40], [62], [79].
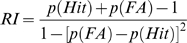
Both SI and RI are based on the same geometric logic and are both appropriate for use with single choice procedures (respond or not; [40]). However, RI does not take into account trials were a subject fails to respond, hence a second bias parameter is needed to ensure alterations in this propensity are properly captured. The perceptual bias measure B″ was calculated to identify the amount of signal required to generate a response [80].
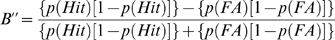



Once fully trained and at asymptote, performance was compared over a three day period (Wednesday, Thursday and Friday) in the standard tasks. The subsequent week, performance was challenged whereby each task continued for 60 min or until 250 trials had been completed. This increased duration/trial number challenge had led to performance differences between groups of mice in the 5CSR task previously [49]. Challenge days (Monday, Wednesday, and Friday) were interspersed with training days (Sunday, Tuesday, and Thursday).

### Statistics

Data analyses were consistent for baseline assessment and challenge day performance. Data obtained for strain comparison within the 5C-CPT were subjected to a repeated measures analysis of variance (ANOVA), with strain as a between subjects factor and day as a within subjects factor. Data obtained from the 1CRT task, 5CSR task, and 5C-CPT comparison studies in C57BL/6J mice were subjected to a repeated measures ANOVA with task as a between subjects factor and day as a within subject factor. Where applicable, *post hoc* analyses of statistically significant main effects were conducted using Tukey tests. The level of probability for statistically significant effects was set at 0.05. Data were analyzed using SPSS (Chicago, U.S.A.).
